# Supplementary material for: Prevalence, risk factors and predicted risk of cardiac events in chronic kidney disease of uncertain aetiology in Sri Lanka: A tubular interstitial nephropathy
Source: PLoS One. 2021 Apr 14;16(4):e0249539. doi: 10.1371/journal.pone.0249539 (PMC8046203; doi:10.1371/journal.pone.0249539)
Supplement: Attachment — Submitted filename: Response to Reviewers 10_11_2020.docx [file pone.0249539.s001.docx]

November 10, 2020

Editor-in-Chief,

PLOS ONE.

Dear Editor:

We write to acknowledge you and the reviewers for the care and effort they have taken in reviewing the manuscript. We have gone through all the points that the reviewers have raised. I hereby attach an annotated version of the full reports of each reviewers so that you can readily follow our responses in context. In addition, we have uploaded our corrected manuscript incorporating responses to the reviewer comments to the journal website.

I hope that you will be satisfied with the outcome and that you will that feel we have done justice to the suggestions from the reviewers. We believe that the manuscript is now suitable for publication in PLOS ONE. Thank you in advance for your continued consideration and support for our manuscript. If you have any further concerns, please feel free to contact me.

Yours sincerely,

Thilini W.Hettiarachchi (B.pharm, PhD),

Center for Education, Research and Training on Kidney Diseases, Faculty of Medicine, University of Peradeniya, Sri Lanka

Office: +94712284737

Email:hatwasana@gmail.com

**Index of Changes in Response to Reviewers’ Comments.**

Journal Requirements:

 We appreciate this feedback from the Editors.

Comment 1: Please ensure that your manuscript meets PLOS ONE's style requirements, including those for file naming. The PLOS ONE style templates can be found at

<https://journals.plos.org/plosone/s/file?id=wjVg/PLOSOne_formatting_sample_main_body.pdf> and

<https://journals.plos.org/plosone/s/file?id=ba62/PLOSOne_formatting_sample_title_authors_affiliations.pdf>

Response:

Complied with the given formats.

Comment 2: Please amend your current ethics statement to address the following concerns:  
a) Did participants provide their written or verbal informed consent to participate in this study?
b) If consent was verbal, please explain i) why written consent was not obtained, ii) how you documented participant consent, and iii) whether the ethics committees/IRB approved this consent procedure.

Response:

Please find the amended ethics statement (lines 106-108).

a). Yes, they have provided written informed consent b). i) No, the consent was not verbal, it is written. ii) Consent was documented in individual consent forms. iii). Yes, ethics committee approved this consent procedure.

**Review Comments to the Author**

**Reviewer #1**: The study addresses an important yet unaddressed topic - Cardiovascular risk in CKDu. The authors outline the study aims as identifying the prevalence and the risk of CVD in patients with CKDu, However, the methods described in the study and sampling approaches would need to address the following.

We were highly impressed by the statement on understanding the importance of this sudsy in an area with limited knowledge.

*Comment 1: Mean duration of hospital-based follow up of patients with CKDu at the study sites as this are likely to have an impact on the findings on the cardiovascular risk profile.*

Response: As reviewer correctly identified these patients were regularly followed up at two renal clinics in the endemic area of CKDu, Sri Lanka. Mean duration of the hospital-based follow up was 6.2 ± 3.4 years (mentioned in the line 182).

*Comment 2: Sample size calculations (taking into consideration the prevalence of CKDu in the endemic regions of Wilgamuwa and Giradurukotte).*

Response: We have invited all the followed-up patients (n=132) with confirmed CKDu (renal biopsy findings compatible) at renal clinic Wilgamuwa and Girandurukotte during the period from July 2016 to end February 2017. Thirteen patients denied the participation. All remaining participants were included as in total purposive sampling. Since all the available patient count is less as 119, no special randomization technique or sample size calculation was used.

*Comment 3: Any attempts to document history of first-degree relatives with cardiovascular events or inherited disorders of lipid metabolism (Familial Hypercholesterolemia).*

Response: We agree with the reviewer importance of familial risk factors of CVD, but unfortunately, we have not documented the history of first-degree relatives with cardiovascular events or dyslipidaemias

*Comment 4: Cardiovascular risk calculation - Prior studies from Sri Lanka have indicated that Framingham Risk / UKPS risk engines are better for CVD risk screening for detecting subclinical Atherosclerosis as compared to the WHO Risk tools (Journal of Clinical and Diagnostic Research: JCDR. 2016 Jul;10(7): OC09.)*

Response: We also had similar concerns in selecting appropriate prediction model. Herath *et al* in 2016 found that CVD risk assessment tools, both United Kingdom Prospective Diabetes Study risk engine and Framingham Risk Score have almost equal ability (former being marginally superior) in predicting underlying atherosclerotic vascular disease in patients with type 2 diabetes mellitus (T2DM). Nevertheless, in this study, we analysed a different population of patients with CKDu, who did not have a history of type 2 diabetes mellitus. Hence, there is no specific tool for cardiovascular risk prediction in CKDu Sri Lanka we used PAHO/WHO (Pan American Health Organization/World Health Organization cardiovascular) Risk Calculator in predicting cardiovascular risk in this specific population, which is more simpler and easily implementable even in resource-poor settings. Meanwhile, we agree a correct validated risk prediction model is required for each specific group of patients for better results.

(Herath H. M., Weerarathna T. P., Dulanjalee R. B., Jayawardana M. R., Edirisingha U. P., Rathnayake M. Association of Risk Estimates of Three Different Cardiovascular Risk Assessment Tools with Carotid Intima Media Thickness in Patients with Type 2 Diabetes. J. Clin. Diag. Res. 2016;10(7) OC09-12)

More details on the following would be useful

*Comment 5: Line 164: All 132 CKDu confirmed subjects - Where these newly confirmed CKDu during the study period at these two referral centres?. It would also be useful to understand the proportion of CKD patient pool from which these were confirmed and if there were any similarities in the baseline risk profiles as the manuscript restricts the analysis to CKDu with or without IHD however draws inferences comparing with CKD in general.*

Response: Total CKD/CKDu patients registered at the two-referral centres (Wilgamuwa and Girandurukotte) was 2094. Only 132 patients were observed with confirmed CKDu; which was determined through a kidney biopsy. (Clarified in lines 180-181 and fig 1). Histopathological features consistent with CKDu on biopsy are the features of (a) Glomerular sclerosis and glomerular collapse, (b) interstitial fibrosis, (c) tubular fibrosis, (d) tubular atrophy, (e) interstitial infiltration, (f) presence of casts, and (h) arteriolar hyalinosis (Selvarajah *et al*., 2016), preferably the demonstration of the absence of immune deposits. Yet, there may be other patients with CKDu; who had denied undergoing biopsy procedures. Therefore, it was not practical to demarcate CKD and confirmed CKDu without kidney biopsy. In that case, we only considered patients with confirmed CKDu, to obtain a better clinical picture of the cardiovascular morbidity in patients with CKDu. Since there is no literature on CKDu related cardiovascular morbidity, we drew inferences comparing with CKD in general. It is useful to compare the baselines of different groups within CKD, but unfortunately, it was not studied and a limitation to generalize our findings.

(Selvarajah M, Weeratunga P, Sivayoganthan S, Rathnatunga N, Rajapakse S. Clinicopathological correlates of chronic kidney disease of unknown etiology in Sri Lanka. Indian J Nephrol. 2016;26(5): 357–363. pmid:27795631)

*Comment 6: Line 193 Table 2: Self-reported IHD, Myocardial Infarction in 5 participants - was this validated with hospital records, discharge summary or EKG/ ECHO findings during the evaluation.*

Response: Yes, all the events of myocardial infarction was validated with discharge summary and hospital records. (Mentioned in the top row in Table 3)

*Comment 7; Line 336: Hyperuricemia showed a substantial risk for IHD. There have been reports on the potential association of Hyperuricemia as a risk for CKDu in the Mesoamerican Nephropathy( Line 341) suggesting that repeated episodes of AKI might be resulting in CKDu. Is the study adequately powered to draw the inference the significant association?*

Response: We also think hyperuricaemia is an important molecule in the pathogenesis and other manifestations of CKDu. We have stated that “Prevalence of hyperuricemia was reported as high in CKDu in Central American studies of Mesoamerican Nephropathy. Similarly in this study, we report a substantial-high percentage; around 40% with hyperuricemia. In addition to that, the presentation of hyperuricemia in this study of Sri Lankan CKDu has a significant association with IHD”. The study is not adequately powered to comment on AKI events. Revised the lines 356-359

*Comment 8: Line 348 17.65%,(n=21) of later onset of diabetes or HbA1c ≥ 6.5% was identified, would be important to establish what proportion of the subjects had HbA1C values qualifying for Prediabetes at the time of CKDu diagnosis?*

Response: At the time of diagnosis, all the patients were reported as not having Diabetes Mellitus either by HbA1c < 6.4% or fasting blood sugar less than 6.9mmol/l, denoting that none of the patients was Diabetic at the time of diagnosis as per MOH diagnostic criteria(explained in lines 192-193, 363). Even though it is very useful to know, the number who were in the prediabetes range at the time of diagnosis was difficult to extract from available records.

*Comment 9: Line 353, The study did not show a similar association between IHD and HbA1c level, neither does the study show any association with respect to an increased BMI between those with IHD in the CKDu and those without IHD ( Table 2, BMI > 23 p =0.686). It might be important to highlight if there are any such differences in among the CKD populations with or without IHD in the Sri Lankan context.*

Response: In the current study of CKDu we did not observe any significant association between IHD and HbA1c level and again IHD and BMI (kg/m2) > 23. We admit that it is much informative if we compare IHD and HbA1c and IHD and BMI in both CKD and CKDu in Sri Lanka. However, there are no reported literature on HbA1c levels or BMI among the CKD populations with or without IHD in the Sri Lankan context. Therefore, such comparisons were impossible in the current study.

*Comment 10: It would be important to also know what proportion of the subjects were on ACE inhibitors, angiotensin receptor blockers, ß blockers, ASA and Statins and duration of being on these as the cardiovascular risk assessments are likely to be impacted by these interventions.*

Response: We have analysed the medications and duration of being on those medications (Explained in the lines 211-214). Of note, there was no significant impact from the medications in the current study (described in lines 230-234).

“Participants (25.2%, n=30) were on antihypertensive medications (angiotensin-converting enzyme inhibitors, angiotensin receptor blockers, calcium blockers, alpha-blockers, beta-blockers or diuretics) for a mean duration of 4.2 ± 2.6 years, 22.7% (n=27) were on lipid-lowering therapy with statins for a mean period of 4.9 ± 3.1 years.

From the patients with IHD, 32.4% (n=11) was on antihypertensives and 23.5% (n=8) was on statins. In the other group, who were without IHD, 22.4% (n=19) on antihypertensives and 22.4% (n=19) on statins. Notably, there was no significant difference between the proportion of patients on each treatment (antihypertensives (p= 0.350) or lipid-lowering agent; statin (p =1.000)) in either group, with IHD or without IHD.”

**Reviewer #2: PLOS ONE**

*This is an interesting piece of work and relevant for clinicians globally caring for patients with CKDu. The question of whether the risk of cardiovascular disease/morbidity in patients with CKDu is the same as those with CKD is important for patients, clinicians and health care providers.
The authors have conducted a study with reasonable scientific rigour, but the writing needs some more work to make the results easy to understand.
The discussion needs to be reframed to focus on the main finding that the rates of cv events are lower and patients are likely to survive to needing treatment for eskd and delaying the onset of eskd is crucial to their management*

We highly acknowledge the comments from the reviewer #2. We have restructured the discussion by focusing on the main findings that rates of CV events are lower and concluding that patients are likely to survive to ESKD and delaying the onset of ESKD is crucial.

*Please see specific comments below*.
*Comment 1: Abstract: Line 30 – I suggest re-write as below; Cardiovascular disease (CVD) is the leading cause of morbidity and mortality in patients with ‘traditional’ chronic kidney disease (CKD. However, chronic kidney disease of uncertain aetiology (CKDu), a tubular interstitial nephropathy is typically minimally proteinuric without high rates of associated hypertension or vascular disease and it is unknown if the rates of cardiovascular disease are similar.*

Response: Admitted and corrected in the lines 29-33

*Comment 2: Line 33 - This study aimed to identify the prevalence and the risk of CVD in patients with CKDu. Delete the rest of the sentence.*

Response: Admitted and corrected in the lines 33-34

*Comment 3: Line 36 remove the word ‘to.’ Instead of the work current use baseline
Perhaps rewrite the sentence starting with in order to –
A detailed medical history, blood pressure, electrocardiogram (resting and six minutes vigorous walking) and echocardiograms, appropriate laboratory parameters and a medical record review was used to collect data at baseline. The WHO/Pan American Health Organization, cardiovascular risk calculator was employed to determine the future risk of CVD.*
Response: Admitted and corrected in the lines 36-40

*Comment 4: Line 41 – rewrite to –
The clinics had recorded xx number of patients with CKDu, of these 119 consented to participation in the study.*
Response: Admitted and corrected in the lines 41-42

*Comment 5: Line 44 – add years*

Response: Done, in the line 44

*Comment 6: Line 49 – add planning for eskd services or something along those lines.*

Response: Corrected as “Our findings highlight the need for developing strategies to minimize the progression of CKDu to end-stage renal disease” (lines 50-51)

***Introduction***

*Comment 7; line 55 – Perhaps say that all stages of CKD are associated with increased cvd risk*

Response: We wanted to elaborate that, even early stages of CKD are associated with increased CVD risk. So we updated the sentence in the lines 54-55.

*Comment 8; Line 57 - ?delete the sentence starting with structural and ….*

Response: Admitted and deleted

*Comment 9: Line 63/Paragraph 2 – should discuss the international findings a little more so its’ more relevant to a global audience such as PLOS one.*

Response: Admitted with thanks. We have discussed the international findings in the lines 58-76

*Comment 10; Line 84 – the sentence starting – in order to …. Is methods – please delete*
Response: Admitted and deleted

***Materials and methods***

*Comment 11; Line 90 -across rather than in*

Response: Corrected ;line 102

*Comment 12: How were patients invited? Need to say somewhere that they were consented and by who.*

Response: Patients were recruited on written informed consent by trained researchers (mentioned in the lines 105-107)

*Comment 13: Line 102 – what do you mean by later onset of diabetes? Subsequent perhaps?*

Response: Yes, Changed the word to subsequent onset of diabetes

*Comment 14: Line 122 – reorder the sentence starting with according to kdigo,
Could just say that the KDIGO definitions were used for stage of ckd and anemia. And reference to a table with all the definitions in the table. Will be easier to read*

Response: Changed the sentence and updated the overall paragraph with definitions for anaemia, hyperparathyroidism and hyperuricemia (in the lines 128-133)

*Comment 15: Line 129 – who assessed the ECGs and was this standardised?*

Response: a cardiologist assessed ECG, based on standard criteria for ECG abnormalities (explained in the lines 135-139 and Table 1). (If the table 1 is over informative, we would like to convert that to a paragraph)

*Comment 16: Who performed the echo’s and was any sort of standard criteria used to record abnormalities?*
Response: An experienced cardiologist performed Echocardiograms and standard criteria was used to record the abnormalities (explained in the lines 135-139 and Table 1). (If the table 1 is over informative, we would like to convert that to a paragraph)

***Results***
*Comment 17: Needs a study flow diagram that shows how many people were invited, how many consented.*

Response: The total CKD/CKDu patients registered at both clinics was 2094. Among them, all confirmed CKDu subjects (132) were invited and 119 were consented to participate in the study. (Flow chart inserted as Fig 1, described in the lines 180-182).

*Comment 18: Line 172 can be removed and table 1 in brackets can be added at the end of the first sentence. Was age normally distributed? If not, best to report medians.*

Response: The line was removed and Table 2 (previous table 1) was cited within brackets in the text (line 195). Age was normally distributed. Therefore, we used mean and standard deviation.

*Comment 19: Line 181 is a key finding and should be given more prominence. It suggests that proteinuria is a negative marker even in a disease not characterised by proteinuria.*

Response: Even the disease is not characterized by heavy proteinuria; number of patients with proteinuria was significantly high in later stages compared to the early stages. (Described in the lines 207-209)
*Comment 20: Line 185 – please re-write, I found it very difficult to understand. I also didn’t understand if the 1 patient (line 187) was from the 119 or out of the 39.*

Response: We have rewritten the paragraph (lines 220-223)

*Comment 21: Table 2 – need a total at the top – was this out of 119 patients or 39 with abnormalities*

Response: Added the total number on top of the table, it is from overall 119

*Comment 22: Line 201 – might be easier to say no incidence of …..*

Response: Corrected in the line 238

*Comment 23: Regarding the figures – it might be helpful for the readers to have some context in terms of the rates of smoking and diabetes in Sri Lanka or among ‘traditional’ CKD patients.*

Response: An epidemiology study carried-out in urban tertiary care centre of Sri Lanka found that the most common underlying causes of CKD were diabetes (88, 44%) and again diabetes was the most common cause of CKD among patients from the western province (74, 54%) (Wijewickrama et al., 2011). However, previous studies have demonstrated that diabetes (2%, 9.6%) contributed to only a minority of CKD/CKDu in the North Central region of Sri Lanka [Athuraliya et al., 2009 and Katulanda et al., 2011]. In the current study of the two CKDu endemic areas of the North Central region observed 17.6% of CKDu patients with the subsequent onset of diabetes. Incidence of smoking was not reported in the settings of CKD, Sri Lanka and the current study of Sri Lankan CKDu, it is 29.4% (Explained in the lines 364-369 and 372-374)

(Wijewickrama ES, Weerasinghe D, Sumathipala PS, Horadagoda C, Lanarolle RD, Sheriff RMH. Epidemiologyof Chronic Kidney Disease in a Sri Lankan Population:Experience of a Tertiary Care Center. Saudi J Kidney DisTranspl 2011; 22: 1289-93.

Athuraliya TNC, Abeysekera DTDJ, Amerasinghe PH, Kumarasiri PVR, Dissanayake V. Prevalence of chronic kidney disease in two tertiary care hospitals: high proportion of cases with uncertain aetiology. Ceylon Med J. 2009;54:23–5.

Katulanda P, Rathnapala DAV, Sheriff R, Mathews DR. Province and ethnic-specific prevalence of diabetes among Sri Lankan adults. Sri Lanka J Diabetes Endocrinol Metabolism. 2011;1:2–7)

*Comment 24: It might be easier to describe this as a group of rhythm abnormalities – easier to read and understand*

Response: Apart from rhythm abnormalities, we have assessed other cardiovascular morbidities like IHD from medical history/clinical records, hospitalization for congestive heart failure, soft tissue vascular calcification. Therefore, we changed the topic as “Prevalence, risk factors and predicted risk of cardiac events in Chronic Kidney Disease of uncertain aetiology in Sri Lanka: a tubular interstitial nephropathy”

*Comment 25: Line 242 – important point – move up;*

Response: Yes, it is important we moved up to top to the lines 255-258

*Comment 26: Table 3 Best to avoid abbreviations as much as possible such as LVH, K, P, Na, Ca HCO3 – use the words instead*

Response: Corrected in table 3 with using the words instead of abbreviations

*Comment 27: Line 272 – instead of overall risk factor better to call it risk calculator or something like that*
Response: Not only the variables from the risk calculator, we have analysed both traditional and non-traditional risk factors, medical history, lifestyle and most biochemical variables, which have probable relationship with cardiovascular disease. Therefore, we would like to keep that section topic as it is.

*Comment 28: Start the paragraph with line 281 rather than the tables.*

Response: Admitted the change in the lines 312-315.

*Comment 29: My earlier comments about abbreviations apply to all the tables*.

Response: Corrected the table using the words instead of abbreviations, where needed.

*Comment 30: The discussion needs to re-written so it is focussed on the main findings of the study. Every finding of the study does not need to be discussed. The key finding is that patients with CKDu have lower risk fo CVD. There is no mention of the outcome of mortality anywhere and that would be useful to discuss and report on in this group. The first 3 paragraphs especially should be re-written.*

Response: We have updated/rewritten the discussion section

*Comment 31: Line 325 – is not was*

Response: Corrected as “CKDu is a tubular interstitial disease among low socio-economic hardworking farmers in rural Sri Lanka” (lines 340-341)

*Comment 32: Line 334 – How common is BMI>23 in Sri Lanka?*

Response: Incidence of BMI>23 in Sri Lanka is vary from area to area. According to the Clinical Guidelines of the Endocrine Society of Sri Lanka, overweight and obesity was considered as BMI; 23 – 24.9 and BMI >25 respectively (Somasundaram et al., 2014). Based on that guidelines we have used BMI>23, which is considered to be overweight and have potential risk for cardiovascular disease.

(Somasundaram N, Rajaratnam H, Wijeyarathne C, Katulanda P, De Silva S, 469 Wickramasinghe, Clinical guidelines: The Endocrine Society of Sri Lanka; Management 470 of obesity. Sri Lanka J Diabetes. 2014;4:55–70)

*Comment 33: Line 356 proteinuria is an important finding and should be given prominence*

Response: Admitted that and explained more in the lines 375-380.
“ Proteinuria plays a significant role in the pathogenesis of IHD [46]. Nevertheless, in this study, the overall prevalence of proteinuria was as low as 26.05%. Hence, it was discovered that proteinuria was a late-stage finding in CKDu compared to early stages. Proteinuria has been associated with graded cardiovascular mortality, acting as risk multipliers across all levels of renal function in CKD (Herzog CA, 2009 and Nagata *et al*., 2013). Contrasting to CKD, proteinuria does not play a significant role in CVD pathogenesis in this specific disease of CKDu.”

(Herzog CA*,* Kidney disease in cardiology., *Nephrol Dial Transplant. 2009 Jan; 24(1):34-7.*

Nagata M, Ninomiya T, Kiyohara Y, et al. EPOCH-JAPAN Research Group Prediction of cardiovascular disease mortality by proteinuria and reduced kidney function: pooled analysis of 39,000 individuals from 7 cohort studies in Japan. Am J Epidemiol. 2013;178(1):1–11)

*Comment 34: The limitations and strengths should be addressed openly.*

Response: Admitted. Explained in the lines 419-428

*Comment 35: Line 365 - please rewrite. I did not understand*

Response: Edited the paragraph (in the lines 414-418)
