# Supplementary material for: Prevalence, risk factors and predicted risk of cardiac events in chronic kidney disease of uncertain aetiology in Sri Lanka: A tubular interstitial nephropathy
Source: PLoS One. 2021 Apr 14;16(4):e0249539. doi: 10.1371/journal.pone.0249539 (PMC8046203; doi:10.1371/journal.pone.0249539)
Supplement: Attachment — Submitted filename: Response to Reviewers 25_01_2021.docx [file pone.0249539.s002.docx]

January 25^th^ , 2021

Editor-in-Chief,

PLOS ONE.

Dear Editor:

We write to acknowledge you and the reviewers for the care and effort they have taken in reviewing the manuscript. We have gone through all the points that the reviewers have raised. I hereby attach an annotated version of the full reports of each reviewers so that you can readily follow our responses in context. Also, we have uploaded our corrected manuscript incorporating responses to the reviewer comments to the journal website.

I hope that you will be satisfied with the outcome and that you will feel we have done justice to the suggestions from the reviewers. We believe that the manuscript is now suitable for publication in PLOS ONE. Thank you in advance for your continued consideration and support for our manuscript. If you have any further concerns, please feel free to contact me.

Yours sincerely,

Thilini W.Hettiarachchi (B.pharm, PhD),

Center for Education, Research and Training on Kidney Diseases, Faculty of Medicine, University of Peradeniya, Sri Lanka

Email:hatwasana@gmail.com

**Index of Changes in Response to Reviewers’ Comments.**

**Title:** Prevalence, risk factors and predicted risk of cardiac events in Chronic Kidney Disease of uncertain aetiology in Sri Lanka: a tubular interstitial nephropathy

**Authors:** Thilini W. Hettiarachchi1, Buddhi N.T.W.Fernando, Thilini Sudeshika, Zeid Badurdeen, Shuchi Anand, Ajith Kularatne, Sulochana Wijetunge, Hemalika T.K. Abeysundara, Nishantha Nanayakkara

**Date:** 25.01.2021

We thank the journal for the opportunity to address the reviewers’ comments, which will improve the paper. We have provided point-by-point responses to the reviewers’ comments below.

**Reviewer #1**:

*Comment 1: Please include the rationale for using the PAHO/WHO (Pan American Health Organization/World Health Organization cardiovascular) Risk Calculator over the other risk prediction tools.*

Authors’ Response: The rationale has been described in our paper (see page 8, lines 163-169, page 20, lines 411 - 419 and page 21, lines 436 - 439). A validated cardiovascular risk prediction tool has not yet been implemented for CKDu; Sri Lanka. Herath *et al* in 2016 found that CVD risk assessment tools, both United Kingdom Prospective Diabetes Study risk engine and Framingham Risk Score have almost equal ability (former being marginally superior) in predicting underlying atherosclerotic vascular disease in patients with type 2 diabetes mellitus (T2DM) [58] . Nevertheless, in this study, we analysed a different population, who did not have a history of T2DM. Therefore, we used PAHO/WHO Risk Calculator, which is simple and easily implementable even in resource-poor settings with the readily available variables (gender, age, tobacco use, systolic blood pressure, diabetes and total cholesterol level) but with the limitation of not being validated for CKDu. Meanwhile, we agree a more appropriate validated risk prediction model is required for this particular group of patients for better results.

**Reviewer #2:** *Abstract*

*Comment 1: The sentence starting The cross-sectional study was conducted on patients with confirmed CKDu who were attending two renal clinics in CKDu endemic-area. Consider re-writing to This cross-sectional study included patients with confirmed CKDu who were attending two renal clinics in CKDu endemic-area*

Authors’ Response: We have revised the abstract and re-written the sentence line 35-36 “This cross-sectional study included patients with confirmed CKDu who were attending two renal clinics in CKDu endemic-area.”

*Comment 2: The sentence starting – left ventricular hypertrophy is missing ‘in’*

Authors’ Response: We have revised the manuscript and corrected the sentence as “Left ventricular hypertrophy was reported in 20.2% (n= 24)” (lines 45-46)

*Comment 3: Line 98 – consider hypothesizing that the risk profile is different to CKD by the study investigators.*

Authors’ Response: We have revised the manuscript and edited the lines 96 to 98 as “Understanding the importance, we designed this study to assess the burden of CVD, risk factors and the risk of developing CVD in CKDu, hypothesizing that the risk profile is different to CKD”.

*Comment 4: How were patients invited?*

Authors’ Response: We have revised the manuscript and included details on how patients invited for the study (see lines 105-108). The following sentences have now been incorporated to the manuscript to improve the clarity. Patients followed up in the routine renal clinics at Girandurukotte and Wilgamuwa hospitals were informed regarding the research in their clinic visit. Thereafter, all patients with the diagnosis of confirmed CKDu were invited to the study by medical officers of the clinic.

*Comment 5: Line 250 – please rewrite this sentence.*

Authors’ Response: We have revised the manuscript and rewritten the sentence as” X-ray images showed soft-tissue vascular calcification in six (5.04%). Further, this vascular calcification was significantly associated (p=0.003, α= 0.05) with lower eGFR." (see lines 250-256)

*Comment 6: Line 334 – please rewrite the sentence – it has been reported.*

Authors’ Response: We have revised the manuscript and re-written the sentence as “At least 35% of patients with CKD, had evidence of an ischaemic event (myocardial infarction or angina) at the time of the presentation to a nephrologist.” (Lines 337-338)

*Comment 7: Line 337 please rewrite the sentence – this lower prevalence of IHD in CKD*

Authors’ Response: We have revised the manuscript and rewrote the sentence as “Furthermore, this finding is compatible with the characteristics of study population including physically active lifestyle, lower BMI, vegetable-based diet, lower prevalence of; diabetes, hypertension and proteinuria.” (Lines 340-342)

*Comment 8: Line 354 – please rewrite the sentence – hence, it attributes to cardiovascular diseases*

Authors’ Response: We have revised the manuscript and reworded the sentence as “Ultimately, the hyperuricemia attributes to cardiovascular diseases” (see lines 357-358)

*Comment 9: Line 360 – please rewrite the sentence – we found…*

Authors’ Response: We have revised the manuscript and rewritten the sentence as “Correspondingly, we observed that elevated Troponin-I was positively correlated with IHD in CKDu” (Lines 364-365)

*Comment 10: Line 381 – ECG is a widely ….*

Authors’ Response: We have revised the manuscript and corrected in the line 385

*Comment 11: Line 382 – Ecgs were used not was.*

Authors’ Response: We have revised the manuscript and corrected in the line 386 as “ECGs were used to identify rhythm abnormalities”
